# Platinum pyrithione induces apoptosis in chronic myeloid leukemia cells resistant to imatinib via DUB inhibition-dependent caspase activation and Bcr-Abl downregulation

**DOI:** 10.1038/cddis.2017.284

**Published:** 2017-07-06

**Authors:** Xiaoying Lan, Chong Zhao, Xin Chen, Peiquan Zhang, Dan Zang, Jinjie Wu, Jinghong Chen, Huidan Long, Li Yang, Hongbiao Huang, Xuejun Wang, Xianping Shi, Jinbao Liu

**Affiliations:** 1 Protein Modification and Degradation Lab, SKLRD, School of Basic Medical Sciences, The affiliated Tumor Hospital of Guangzhou Medical University, Guangdong 511436, China; 2 Division of Basic Biomedical Sciences, Sanford School of Medicine of the University of South Dakota, Vermillion, SD 57069, USA

## Abstract

Chronic myelogenous leukemia (CML) is characterized by the chimeric tyrosine kinase Bcr-Abl. T315I Bcr-Abl is the most notorious point mutation to elicit acquired resistance to imatinib (IM), leading to poor prognosis. Therefore, it is urgent to search for additional approaches and targeting strategies to overcome IM resistance. We recently reported that platinum pyrithione (PtPT) potently inhibits the ubiquitin–proteasome system (UPS) via targeting the 26 S proteasome-associated deubiquitinases (DUBs), without effecting on the 20 S proteasome. Here we further report that (i) PtPT induces apoptosis in Bcr-Abl wild-type and Bcr-Abl-T315I mutation cells including the primary mononuclear cells from CML patients clinically resistant to IM, as well as inhibits the growth of IM-resistant Bcr-Abl-T315I xenografts *in vivo*; (ii) PtPT downregulates Bcr-Abl level through restraining Bcr-Abl transcription, and decreasing Bcr-Abl protein mediated by DUBs inhibition-induced caspase activation; (iii) UPS inhibition is required for PtPT-induced caspase activation and cell apoptosis. These findings support that PtPT overcomes IM resistance through both Bcr-Abl-dependent and -independent mechanisms. We conclude that PtPT can be a lead compound for further drug development to overcome imatinib resistance in CML patients.

Chronic myelogenous leukemia (CML) is a myeloproliferative hematologic neoplasm associated with a t(9;22) chromosomal translocation that gives rise to the Philadelphia (Ph) chromosome and the fusion *Bcr-Abl* gene, which encodes a Bcr-Abl protein with enhanced tyrosine kinase activity.^1, 2^ Bcr-Abl is able to activate a wide range of mitogenic signaling pathways such as MAPK/ERK cascade, PI3K/Akt/mTOR and STATs pathways.^3, 4, 5^ The activation of these pathways in Bcr-Abl-expressing cells results in increased activation and/or expression of a series of anti-apoptotic proteins such as Bcl-2and XIAP, thereby conferring cell survival advantage.^6, 7, 8^ Imatinib is a well-established small molecule tyrosine kinase inhibitor (TKI) that specifically targets the ATP-binding site of Bcr-Abl and thereby prevents the Bcr-Abl autophosphorylation; andit has shown significant efficacy in clinical treatment of CML by inducing cytogenetic and molecular remission.^9, 10, 11^ Despite the specific and remarkable effect of imatinib, an increasing number of CML patients resistant to imatinib are emerging in clinic.^12, 13^ The frequent cause of the imatinib resistance is Bcr-Abl amplification and point mutations in the Bcr-Abl relevant domains.^14, 15, 16, 17^ There are more than 100 reported mutations,^18^ of which most can be conquered by the second-generation tyrosine kinase inhibitors (e.g., nilotinib, dasatinib and bosutinib),^19, 20, 21^ with the exception of the T315I mutation, the most stubborn point mutation, which accounts for about 20% of mutations within the Abl kinase domain.^18^ Ponatinib, as a third-generation of tyrosine kinase inhibitor, has shown activity against refractory CML including those harboring T315I Bcr-Abl.^22^ However, the response in advanced patients is limited because successive use of TKIs leads to the evolution of compounded Bcr-Abl kinase domain mutations that show resistance even to ponatinib.^23^ In addition, the long-term benefit of ponatinib has to be balanced against the risk of deleterious side effects in these patients. Hence, the challenge of overcoming resistance to IM therapy persists in the management of CML.

With the growing understanding of the dependency of cancer cells on a functioning ubiquitin–proteasome system (UPS), and the success in clinical use of proteasome inhibitors (e.g., bortezomib, carfilzomib) to treat multiple myeloma and mantle cell lymphoma, the UPS has proven to be an attractive target for development of drugs for cancer therapy.^24, 25^ Deubiquitinating enzymes (DUBs), a critical component of the UPS, are responsible for removal of ubiquitin monomers and chains before proteasomal degradation and have been implicated in the pathogenesis of cancer.^26, 27^ Members of the DUB family have been shown to be differentially expressed and activated in a number of cancer settings, including CML, with their aberrant activity linked to cancer prognosis and clinical outcome.^28,29,30^ Studies have previously shown that inhibition of proteasomal cysteine DUB enzymes (e.g., USP14 and UCHL5) can be predicted to be particularly cytotoxic to tumor cells as it leads to blocking of proteasome function and accumulation of proteasomal substrates.^31, 32^ Although proteasome inhibitors such as bortezomib and gambogic acid have been reported to downregulate Bcr-Abl expression and induce apoptosis in CML cells,^33, 34^ the study on the effect of DUB inhibitors on Bcr-Abl hematopoietic malignancies is also warranted.

Only recently we have defined that a new platinum-based antitumor agent platinum pyrithione (PtPT), the platinum ion and PT-chelating product has inhibitory activity of 26 S proteasome-associated DUBs and thereby exerts safer and potent antitumor effects.^35^ In the present study, we investigated the antineoplastic effects of PtPT on Bcr-Abl wild-type and Bcr-Abl-T315I mutant cell lines, primary cells from CML patients and mouse IM-resistant xenograft models. Here, we show that PtPT-induced UPS inhibition leads to caspase-3-mediated onset of apoptosis in both IM-resistant and IM-sensitive CML cells and that both UPS inhibition and caspase activation are required for PtPT to induce Bcr-Abl downregulation.

## Results

### PtPT induces proteasome inhibition in CML cells

**Figure 1:**
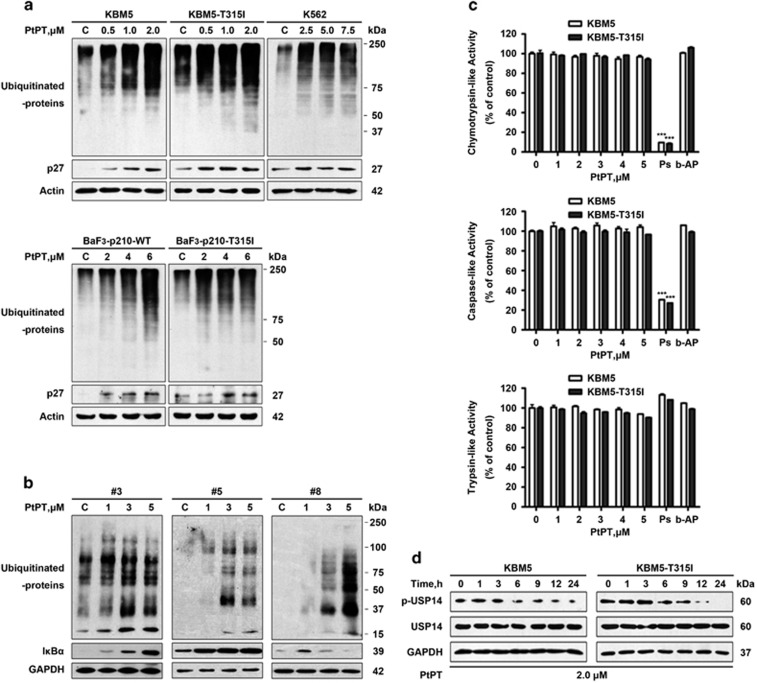
PtPT inhibits proteasome function in CML cells. (**a**) PtPT induces accumulation of polyubiquitinated proteins in CML cells. The cells were treated with the indicated dose of PtPT for 6 h; the protein levels of ubiquitinated proteins (Ubs) and p27 were detected using western blots. (**b**) PtPT accumulates ubiquitinated proteins and I*κ*B-*α* in CML cancer cells. Cancer cells from three CML patients were treated with PtPT for 9 h. The indicated proteins were analyzed using western blots (#3, 5: imatinib-sensitive patients; #8: imatinib-resistant patient). (**c**) PtPT does not inhibit 20 S proteasome peptidase activities in KBM5 and KBM5R cells. Cell lysate was treated with PtPT, and then the chymotrypsin-like, caspase-like and trypsin-like activity at different times were recorded using the fluorogenic Suc-LLVY-AMC, Z-LLE-AMC and Boc-LRR-AMC substrate, respectively. PS341 (Ps) was used as a positive control, and b-Ap15 (b-AP) was used as a DUB inhibitor control. Results shown are the mean of a representative experiment performed in triplicate. ***P<0.001, *versus* the control (0 *μ*M PtPT) group. (**d**) PtPT inhibits the phosphorylation of USP14. KBM5 and KBM5R cells were treated with 2.0 *μ*M PtPT for the indicated duration. Phosphorylated (p-USP14) and total USP14 were detected with western blot

It is well established that inhibition of the proteasome or DUBs causes accumulation of ubiquitinated proteins.^36^ Like what we previously reported with other cancer cells,^35^ PtPT dose- and time-dependently induced marked increases in both ubiquitinated proteins (Ubs) and proteasome substrate protein p27 in all the CML cell lines we tested (Figure1a). To further evaluate the proteasome-inhibiting effects of PtPT, bone marrow cells from 10 patients with CML (3 patients are IM resistant) were treated *ex vivo* with escalating doses of PtPT. PtPT treatment induced marked accumulation of ubiquitinated proteins and proteasome substrate protein I*κ*B-*α* (Figure 1b). Similar to the DUB inhibitor b-AP15, PtPT treatment caused no decline of proteasome peptidase activities (chymotrypsin-like, caspase-like and trypsin-like activity) in KBM5 and KBM5R cells, whereas the proteasome inhibitor bortezomib substantially inhibited the proteasome chymotrypsin-like and caspase-like activity as expected (Figure 1c). These results suggest that as a DUB inhibitor, PtPT does not directly block 20 S proteasome peptidase activity in CML cells, consistent with our previous report. Furthermore, the phosphorylation of USP14 (S241) was substantially reduced in PtPT-treated CML cells (Figure 1d), also indicative of suppression of the DUB activity of USP14 by PtPT treatment.^37^

### PtPT downregulates Bcr-Abl protein and inhibits its downstream signaling

**Figure 2:**
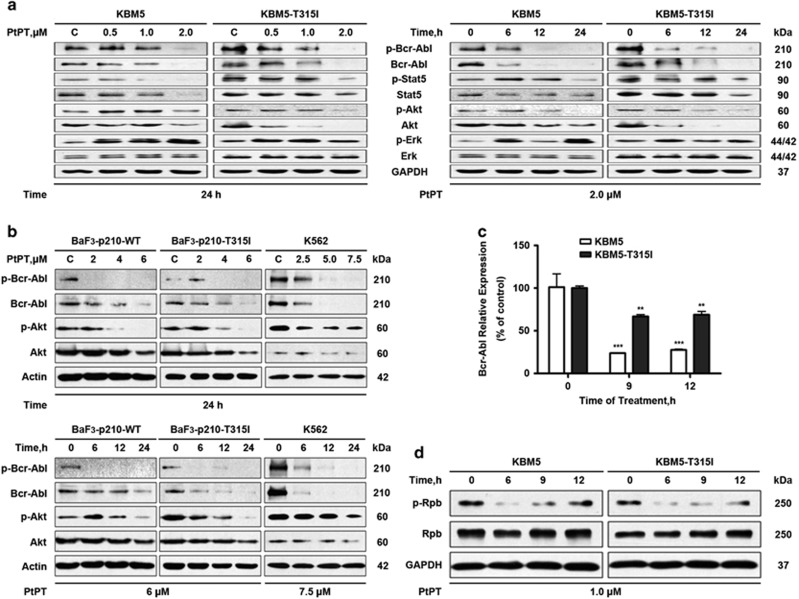
PtPT treatment downregulates Bcr-Abl and its downstream signaling proteins. (**a** and **b**) PtPT decreases the protein levels of Bcr-Abl and its downstream targets in a dose- and time-dependent manner. CML cells were treated with PtPT as indicated, then collected for western blot analyses for the indicated proteins. (**c**) PtPT decreases mRNA expression of Bcr-Abl. KBM5 and KBM5R cells were exposed to 1.0 *μ*M PtPT for 9 or 12 h. The Bcr-Abl mRNA level was determined using RT^2^-PCR and its expression level relative to the control was calculated. Mean±S.D. (*n*=3). **P<0.01, ***P<0.001, *versus* control group. (**d**) PtPT inhibits cellular activity of RNA pol II. KBM5 and KBM5R cells were treated with PtPT (1 *μ*M) for the indicated duration, the phosphorylated (p-Rpb) and total protein levels of RNA pol II (Rpb) were analyzed with western blot

We next determined whether PtPT is capable of inhibiting Bcr-Abl in CML cells. Toward this end, both Bcr-Abl-WT and Bcr-Abl-T315I cells were exposed to increasing concentrations of PtPT for various time periods, the Bcr-Abl and its downstream targets were detected. Our result indicated that PtPT downregulated the levels of the total and phosphorylated Bcr-Abl proteins in both Bcr-Abl-WT and Bcr-Abl-T315I cells in a dose- and time-dependent manner (Figures 2a and b). Further examination displayed that PtPT affected the expression of Bcr-Abl downstream target proteins. The phosphorylation of STAT5 and Akt was also significantly decreased in a dose- and time-dependent manner. Interestingly, PtPT treatment also stimulated phosphorylation of ERK, which may be associated with the catalytic process of autophagy in CML cells, similar to the function of PS341 reported previously.^38^

### Bcr-Abl downregulation results from diminished gene expression

To explore the mechanism of PtPT-mediated decrease in Bcr-Abl, we first detect the transcription of Bcr-Abl with RT^2^-PCR. KBM5 and KBM5R cells were treated with 1.0 *μ*M PtPT for 9 and 12 h, and the mRNA level of Bcr-Abl was decreased to some extent in both cells (Figure 2c). To further address this issue, the phosphorylation of RNA polymerase II was analyzed. As shown in Figure 2d, PtPT leads to inhibition of RNA polymerase regardless of the mutation status of the *Bcr-Abl* gene. Although the degree of mRNA reduction is apparently less than the reduction of the corresponding protein levels, the downregulation of mRNA by PtPT likely contributes to the decrease of the Bcr-Abl proteins.

### PtPT inhibits growth of both IM-sensitive and IM-resistant CML cells

**Figure 3:**
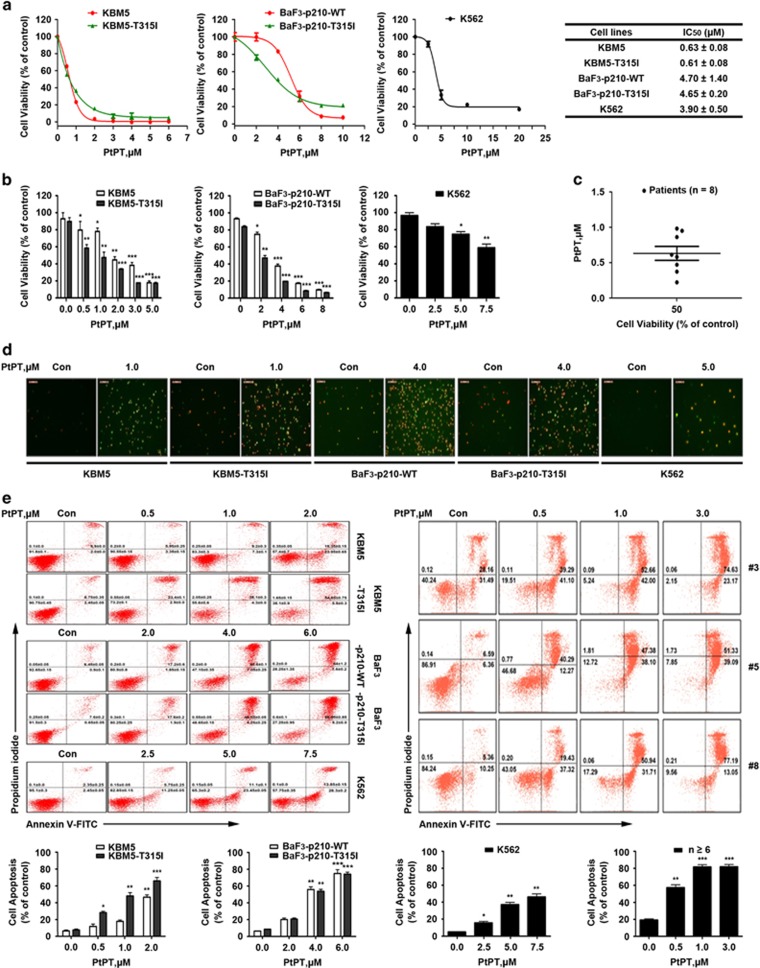
PtPT inhibits cell viability in CML cell lines. (**a** and **b**) PtPT decreases the cell viability of both IM-sensitive and IM-resistant CML cell lines. KBM5, KBM5R, K562, BaF3-p210-WT and BaF3-p210-T315I cells were treated with the indicated concentrations of PtPT for 48 h, and then were subject to MTS assays (**a**). Cell viability was also examined by trypan blue exclusion staining assay. After treatment with designated concentrations of PtPT, CML cells were analyzed with Trypan blue staining for the viability changes (**b**). Graphs represent data from three repeats. Mean±S.D. (*n*=3). *P<0.05, **P<0.01, ***P<0.001, *versus* control group. (**c**) PtPT decreases cell viability of CML patient cancer cells. CML cells from 10 CML patients were treated with PtPT at the indicated doses for 48 h, and cell viability was detected by the MTS assay. Mean±S.D. (*n*=3). (**d** and **e**) PtPT induces cell apoptosis in CML cells. Cells (including mononuclear cells from CML patients) were exposed to the indicated dose of PtPT for 24 h, and cell apoptosis was observed by recording the Annexin V-FITC/PI-positive cells under an inverted fluorescence microscope (**d**) (one of doses with PtPT was shown, respectively) or detected by Annexin V-FITC/PI double staining with flow cytometry (**e**). Mean±S.D. (*n*=3). Representative images were shown. *P<0.05, **P<0.01, ***P<0.001, *versus* control group

We first analyzed the effect of PtPT on growth in IM-sensitive and IM-resistant CML cells. Both Bcr-Abl wild-type IM-sensitive cell lines (KBM5, K562 and BaF3-p210-WT) and Bcr-Abl-T315I mutated IM-resistant cell lines (KBM5R and BaF3-p210-T315I) were treated with escalating concentrations of PtPT for 48 h, and then cell viability was assessed by the MTS assay. PtPT was capable of inhibiting the growth of all the cell lines (Figure 3a). The effect of the compound on cell viability was further examined in CML cell lines by Trypan blue exclusion staining assay. As shown in Figure 3b, a time- and dose-dependent inhibition of cell viability was observed, reflecting its anti-leukemia activity. As displayed in Figure 3c, PtPT decreased the cell viability of primary monocytes from CML patients with IC_50_ values ranging from 0.22 *μ*M to 1.10 *μ*M (0.68±0.3 *μ*M), while for the mononuclear cells from six healthy volunteers, the IC_50_ values were more than 4.4 *μ*M as we reported recently, approximately 6.5 times higher than that for primary monocytes obtained from CML patients.^27^

### PtPT induces apoptosis in both IM-sensitive and IM-resistant CML cells

A dose-dependent proapoptotic effect of PtPT was confirmed by recording the number of Annexin V/PI-positive cells under an inverted fluorescence microscope, and flow cytometry analysis after dual staining of Annexin V-FITC/PI in both Bcr-Abl wild-type and T315I mutant cell lines (Figures 3d and e, left). In addition, we found that PtPT treatment at doses from 0.5 to 3.0 *μ*M for 36 h resulted in significant apoptosis in all the primary monocytes from the 10 CML patients as detected with Annexin V/PI double staining (Figure 3e, right).

**Figure 4:**
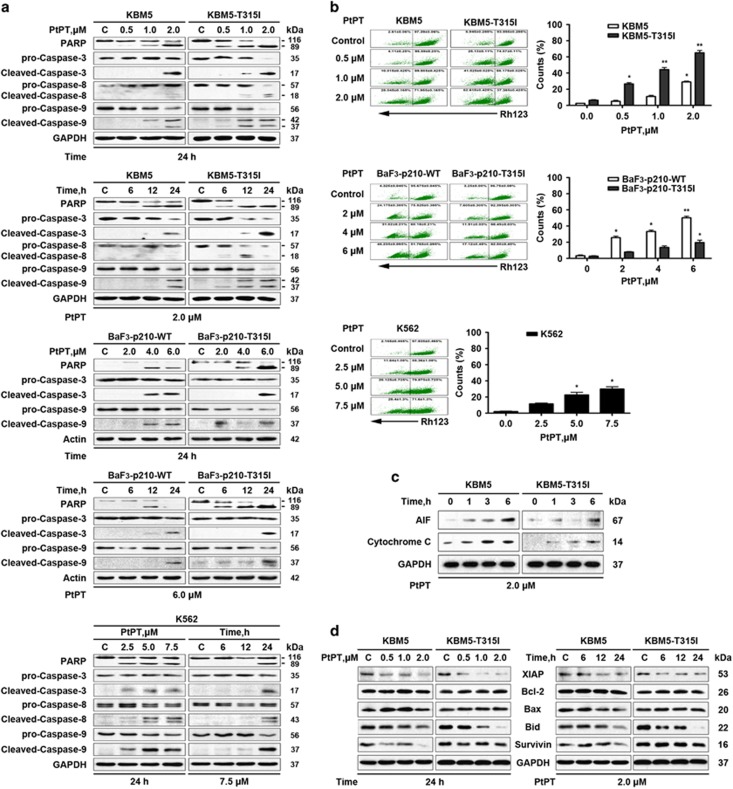
PtPT induces caspase-dependent cell apoptosis. (**a**) PtPT treatment activates caspase. KBM5, KBM5R, K562, BaF3-p210-WT and BaF3-p210-T315I cells were exposed to PtPT at the indicated dose and for the indicated duration, followed by detection of the cleavage of PARP, caspase-3, -8 and 9. (**b**) PtPT reduces mitochondrial membrane potential in CML cells. Cells were treated with various doses of PtPT for 24 h, and then mitochondrial membrane potential was evaluated by rhodamine-123 staining coupled flow cytometry. Mean±S.D. (*n*=3). *P<0.05, **P<0.01, *versus* control group. (**c**) PtPT induces the release of cytochrome C and AIF in CML cells. KBM5 and KBM5R cells were exposed to 2.0 *μ*M PtPT for 1, 3 and 6 h, the cytosolic protein levels of cytochrome *c* and AIF were analyzed with immunoblotting. (**d**) PtPT downregulates anti-apoptotic proteins in CML cells. KBM5 and KBM5R cells were treated with the indicted doses of PtPT for the indicated duration; cell lysates were then immunoblotted to assess the changes of XIAP, Bcl-2, Bax, Bid and Survivin. GAPDH was used as a loading control

We next reasoned that the expression of PARP, as well as caspase-3, -8 and -9 might be affected by incubation with PtPT. Indeed, PtPT induced a dose- and time-dependent specific cleavage of PARP and caspase-3, -8 and -9, all hallmarks of apoptosis (Figure 4a).

It is a widely accepted concept that mitochondrion is the regulating center of apoptosis. Our result showed that the integrity of mitochondrial membranes was decreased in all the CML cell lines treated with PtPT (Figure 4b). The release of cytochrome *c* and apoptosis induce factor (AIF) from mitochondria to the cytoplasm has been recognized as the early signs of apoptosis.^39^ To clarify the involvement of the apoptosis pathway triggered by PtPT, we treated KBM5 and KBM5R cells for different durations and then determined cytochrome *c* and AIF in the cytosolic fractions. As displayed in Figure 4c, cytochrome *c* and AIF release elevated at earlier time points, indicating that PtPT induced mitochondrial pathway of apoptosis in CML cells. Meanwhile, PtPT treatment significantly reduced the levels of pro-caspase-3, and induced PARP cleavage in the primary monocytes (Figure 4d).

To further investigate the mechanism of apoptosis induced by PtPT, we examined the expression of other apoptosis-related proteins. Results showed that there was a decline of anti-apoptotic proteins XIAP and survivin, and of Bid, a mediator of mitochondrial damage induced by caspase-8 (Figure 4d).

### PtPT triggers the endoplasmic reticulum stress response in CML cells

**Figure 5:**
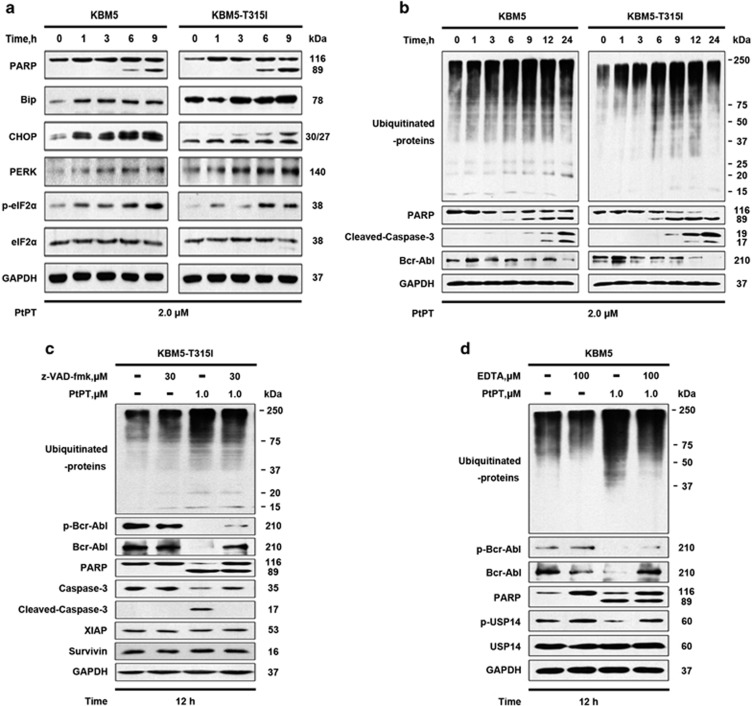
PtPT treatment induces Bcr-Abl downregulation via inhibiting proteasome activity in CML cell lines. (**a**) PtPT treatment induces ER-stress response in KBM5 and KBM5R cells. Cells were treated with 2.0 *μ*M PtPT for the indicated duration, then cell lysates were immunoblotted to assess the changes of PARP, Bip, CHOP, PERK, p-eIF2*α* and eIF2*α*. (**b**) Proteasome inhibition-mediated caspase activation induces Bcr-Abl downregulation. KBM5 and KBM5R cells were treated with 2.0 *μ*M PtPT for the indicated durations. The Ubs, PARP, cleaved-caspase-3 and Bcr-Abl were analyzed with western blot. Representative images are shown. (**c**) PtPT decreases Bcr-Abl and the downstream signaling proteins in a caspase-dependent manner. KBM5R cells were treated with 1.0 *μ*M PtPT for 12 h in the absence or presence of 30 *μ*M pan-caspase inhibitor z-VAD-fmk. The total and phosphorylated Bcr-Abl, Ubs, PARP, caspase-3, cleaved-caspase-3,XIAP and Survivin were analyzed using western blots. Representative images are shown. (**d**) Effect of EDTA on PtPT-induced proteasome inhibition, apoptosis and Bcr-Abl downregulation. KBM5R cells were incubated with 1.0 *μ*M PtPT in the absence or presence of 100 *μ*M EDTA for 12 h, and then Ubs, p-Bcr-Abl, Bcr-Abl, PARP, p-USP14 and USP14 proteins were detected using western blot. GAPDH was used as a loading control

UPS inhibition induces unfolded protein response (UPR) and apoptosis.^40^ Examination of the effect of PtPT on the endoplasmic reticulum (ER) stress response showed that treatment of both KBM5 and KBM5R cells with PtPT activates PERK-mediated ER stress signaling, evidenced by significant induction of p-eIF2a. In addition, PtPT treatment triggered induction of CHOP and Bip (Figure 5a). Together, these results suggest that PtPT-mediated proteasome inhibition has an important role in PtPT-induced ER stress response in CML cells.

### Bcr-Abl downregulation results from caspase-dependent cleavage

We and others have reported that the caspase activation pathway is involved in the degradation of Bcr-Abl.^34, 41^ We thus hypothesized that the caspase activation might be involved in the PtPT-induced Bcr-Abl decline. To test this hypothesis, KBM5R cells were incubated with PtPT in the absence or presence of the pan-caspase inhibitor z-VAD-fmk, and Bcr-Abl proteins were assessed. Specifically, we observed that z-VAD-fmk mostly blocked PtPT from inducing cell death and from decreasing Bcr-Abl proteins to a certain extent but showed no effect on ubiquitinated protein accumulation (Figures 5b and c). These results demonstrate that caspase activation triggered by PtPT-induced proteasome inhibition contributes to the downregulation of Bcr-Abl.

### Proteasome inhibition is a critical mediator for PtPT-induced apoptosis

Accumulation of ubiquitinated proteins, as well as the decrease of phosphorylated USP14 were observed as early as 6 h during the course of treatment (Figures 1a and d). Importantly, obvious apoptosis-specific PARP cleavage was not observed until 9 h of PtPT treatment (Figures 4a and 5b). These results show that the apoptosis induced by PtPT occurs after the proteasome inhibition. To further address this issue, we tested the effect of ethylenediaminetetraacetic acid (EDTA), a chelating agent for metal ions such as Pt^2+^, on the PtPT induction of proteasome inhibition and cell apoptosis. The result shows that PtPT exhibited diminished reactivity after being bound by EDTA. As expected, EDTA partially reversed PtPT-induced ubiquitinated protein accumulation and phosphorylated USP14 downregulation; PARP cleavage was accordingly abolished; and the decreases of Bcr-Abl proteins was apparently recovered in KBM5R cells (Figure 5d). Therefore, proteasome inhibition is required for cell apoptosis induction by PtPT.

### PtPT inhibits the tumor development from the xenografted Bcr-Abl-WT and -T315I mutant cells in nude mice

**Figure 6:**
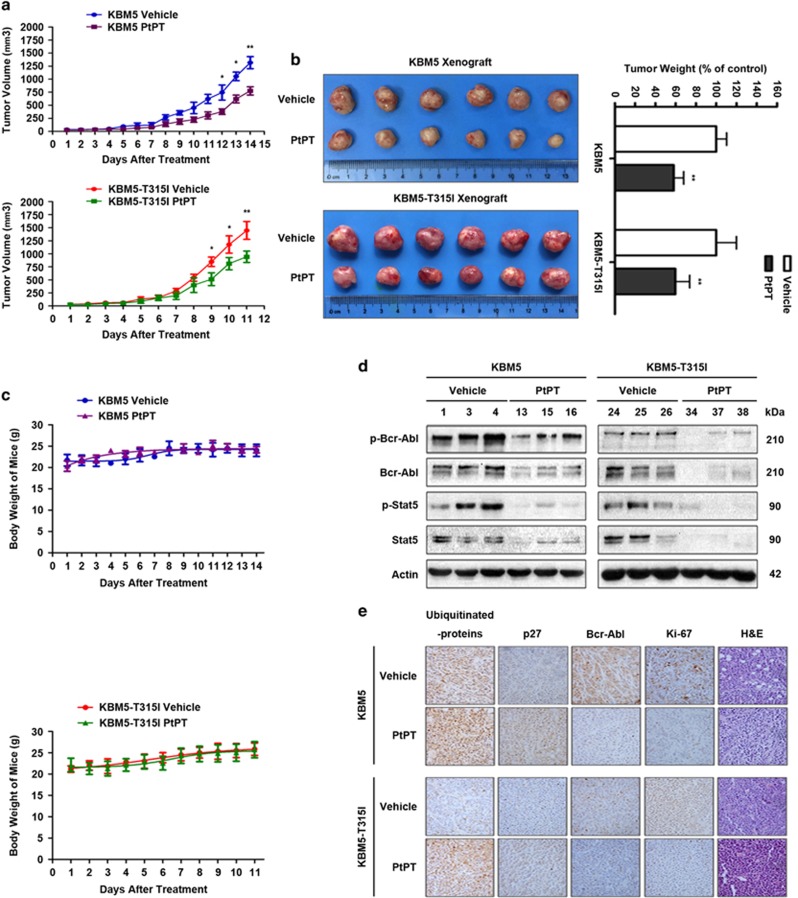
*In vivo* effect of PtPT on KBM5 and KBM5R cells derived mouse xenograft models. Nude mice bearing wild-type and T315I-mutant Bcr-Abl xenograft tumors were treated with either vehicle or PtPT (7 mg/kg/day) for 11–14 days after inoculation of KBM5 and KBM5R cells. (**a**) PtPT inhibits tumor growth *in vivo*. Tumor growth curves were recorded every day in two sets of experiments. Mean±S.D. (*n*=6). *P<0.05, **P<0.01, *versus* PtPT-treated group. (**b**) On day 11 or day 14 after inoculation, the mice were killed, and the tumor tissues were weighed and imaged. **P<0.01, *versus* control group. (**c**) The mice weights were recorded every day after PtPT treatment. Mean±S.D. (**d** and **e**) Western blot analyses (**d**) and/or immunohistochemistry staining (**e**) for proteasome substrates (ubiquitinated proteins, p27), Bcr-Abl-related proteins (Bcr-Abl, p-Bcr-Abl) and its downstream proteins (Sta5, p-Stat5) and protein biomarker related to proliferation (Ki-67) in the xenograft tumor tissues from the KBM5 vehicle group (#1, 3, 4), KBM5 PtPT-treated group (#13, 15, 16), KBM5-T315I vehicle group (#24, 25, 26) and KBM5R-T315I PtPT-treated group (#34, 37 38). In **e**, the proteins of interest are immunostained brown. All the immunohistochemical staining were repeated in three mouse tumor tissues and the representative images are shown

We further evaluated the *in vivo* effect of PtPT on Bcr-Abl-WT and Bcr-Abl-T315I cells using the nude mouse xenograft model. KBM5 and KMB5R cells were inoculated subcutaneously in nude mice. When the size of tumor reached ~50 mm^3^ (3 days after inoculation), the mice were randomized to receive treatment with vehicle or PtPT (7 mg/kg/day) for 11–14 days. A markedly smaller tumor size was noted in PtPT-treated mice *versus* mice receiving vehicle alone (Figures 6a and b) while the body weight remained relatively stable in each group (Figure 6c). Motor activity and feeding behavior were all normal (data not shown). Immunoblotting of xenograft tissues from mice demonstrated that PtPT potently inhibited Bcr-Abl and its downstream pathways (Figure 6d). Immunohistochemical analysis revealed that the ubiquitinated proteins and proteasome substrates p27 were highly accumulated in PtPT-treated tumor tissues *versus* the vehicle-treated models (Figure 6e), indicating that PtPT inhibits proteasome function in both IM-sensitive and -resistant xenografts. The protein biomarker related to cell proliferation, Ki-67, was also downregulated by the PtPT-treatment in both KBM5 and KBM5R models (Figure 6e). Together, these data demonstrate the *in vivo* antitumor activity of PtPT against CML cells regardless of its Bcr-Abl-T315I mutation status.

## Discussion

Despite the success of imatinib in treatment of Bcr-Abl-positive CML, the development of resistance to imatinib, especially for those in accelerated and blast phases, remains a major challenge for treatments.^12, 14^ To overcome this resistance, second-generation TKIs (such as nilotinib, dasatinib and bosutinib) have been developed and are effective against a range of Bcr-Abl mutations (e.g., E255K, M351T) except T315I.^19, 20, 21^ In addition, the resistance to the third-generation TKIs ponatinib would limit the application of the agent to a certain extent.^23, 42^ An approach to tackling this challenge is to find novel drug targets and additional means to interrupt Bcr-Abl signaling pathways. With the growing understanding of the molecular biology of the UPS, proteasome and DUB inhibition have proven to be attractive strategies for cancer therapy.^27, 32^ Importantly, in contrast to 20 S proteasome inhibitor, most of reported DUB inhibitors can specifically target one or several DUBs by which more specific and less toxic anticancer agents may be generated. The potential to influence processes such as signal transduction, proliferation and apoptosis by affecting deubiquitination and proteasomal degradation of key regulators is both promising and exciting. Additional compounds that induce DUB inhibition-dependent apoptosis of CML cells with limited general toxicity are still needed.

In the previous study, we have reported that PtPT synthesized in our lab could produce tumor tissue-specific inhibition of proteasome-associated DUBs and tumor-specific toxicity, showing clinical significance for designing novel strategies for cancer treatment.^35^ Here, consistent with findings derived from studies using other cancer cells, PtPT induced UPS inhibition in both Bcr-Abl wild-type and Bcr-Abl T315I cell lines, as well as in primary mononuclear cancer cells derived from CML patients including IM-sensitive and -resistant cells. It was further confirmed that PtPT also inhibited UPS function in the xenografted tumor model bearing wild-type- and T315I *Bcr-Abl* genes *in vivo*. More importantly, we analyzed the mechanism of action of PtPT in overcoming IM-resistant cells. In the *in vitro* study, PtPT dose- and time-dependently causes significant mitochondrial damage and apoptosis in both IM-sensitive and IM-resistant CML cells; in the mononuclear cancer cells from CML patients, PtPT also decreased cell viability and induced cell death; in the *in vivo* experiment, the growth of both IM-sensitive and -resistant xenografted tumor cells were inhibited by PtPT treatment. Taken together, PtPT could be a potential candidate for treatment of imatinib-resistant CML. To our knowledge, this is the first report to show that PtPT, as a novel inhibitor of 26 S proteasome-associated DUBs, is effective against CML cells, including those harboring gate-keeper mutant T315I Bcr-Abl.

Although further studies are warranted to determine the mechanism by which PtPT induces cell apoptosis and overcomes IM resistance, both Bcr-Abl-dependent and -independent mechanisms have been documented to modulate the expression of Bcr-Abl. On one hand, PtPT inhibits the transcription of the Bcr-Abl, and future studies need to investigate whether the diminished *Bcr-Abl* gene expression is *Bcr-Abl* gene-specific or a general gene transcription inhibition. On the other hand, PtPT-induced UPS inhibition-dependent caspase activation cleaves Bcr-Abl (Figures 5b and d), then leading to downregulation of Bcr-Abl and inhibition of cell proliferation. Here, our findings uncover an exciting new mechanism that PtPT-mediated UPS inhibition and caspase activation are capable of downregulation of Bcr-Abl protein; the latter contributes to PtPT’s overcoming IM resistance in CML cells.

We have reported that proteasome inhibition-induced ER stress response has an important role in caspase activation and cell apoptosis.^43^ Here we also found that PtPT time-dependently induced ER stress-response pathway (Figure 5a). We speculate that the induced ER stress response contributes to the induction of mitochondrial damage, which is reflected on the increasing release of cytochrome *c* and the caspase activation (Figure 4). Activated caspases, induce PARP cleavage and apoptosis on one hand and directly cleave Bcr-Abl proteins on the other.

In addition, our findings revealed that PtPT induced decreases in Bcr-Abl levels and its downstream targets (i.e., STAT5, Akt). Given that Bcr-Abl is an addiction oncogene in CML cells, lowering Bcr-Abl level and thereby disabling its signaling may result in cell growth inhibition. It is worthy to note that PtPT distinctly triggers ERK phosphorylation, although it is not sufficient to fully protect CML cells from DUBs inhibition-mediated apoptosis. PtPT-treatment stimulated ERK phosphorylation may attribute to the catalytic process of autophagy in leukemia cells, just as the action of bortezomib previously reported.^38^ In this regard, PtPT-mediated autophagy process may enhance the chemotherapy efficacy of the agent. It also remains to be investigated whether PtPT is effective in the CML stem cells, which is believed to be a critical cause of imatinib resistance and an obstacle to curing CML.

CML, being highly dependent on presence of Bcr-Abl, is a classical model of oncogene addiction.^44^ Inactivation of Bcr-Abl kinase by TKIs (e.g., imatinib, dasatinib) or downregulating the total levels of cellular Bcr-Abl by triptolide (at the mRNA level),^41^ gambogic acid,^34^ auranofin^45^ and PtPT^35^ (at the protein level) could be effective approaches to block the 'addiction' in Bcr-Abl-expressing cells. Although triptolide, gambogic acid, auranofin and PtPT may impact on multiple molecules, Bcr-Abl downregulation is the common effect and is likely the major factor overcoming IM resistance in Bcr-Abl-expressing cells. At present, most studies on Bcr-Abl inhibition are centered in finding TKIs that directly inhibit tyrosine kinase activity. Here we propose an alternative strategy to enhance Bcr-Abl cleavage by activating the caspase system via DUB inhibition.

Collectively, our *in vitro*, *ex vivo* and *in vivo* results demonstrate that PtPT has potent activity against CML cells carrying wild-type or Bcr-Abl-T315I mutation. Moreover, DUB inhibition-mediated caspase activation and Bcr-Abl downregulation are responsible for the proapoptotic effects of PtPT on Bcr-Abl-positive cells. Taken together, PtPT may have clinical efficacy against human CML driven by activated Bcr-Abl regardless of the mutation status, providing great importance in future clinical CML therapy, particularly in those suffering from imatinib resistance.

## Materials and methods

### Cell culture

KBM5 cells bearing 210 kDa wild-type Bcr-Abl were derived from a female myeloid CML patient in blast crisis as described previously.^41^ KBM5-T315I cell line (KBM5R), an imatinib-resistant KBM5 subline, was derived from KBM5 by exposing to increasing concentrations of imatinib, and subsequent selection of the survival clone harboring T315I mutation as previously described.^34, 41^ Both of these cell lines were cultured in Iscove's modified Dulbecco's medium (Gibco-BRL, Gaithersburg, MD, USA) supplemented with 10% fetal calf serum (FCS), as described previously. In addition, the KBM5-T315I cells were also grown with 1.0 *μ*M imatinib. Imatinib was washed off and KBM5-T315I cells were cultured in drug-free medium for several days before the experiments. The murine BaF3 cells stably expressing either 210 kDa wild-type Bcr-Abl (BaF3-p210-WT) or T315I Bcr-Abl (BaF3-p210-T315I) were kindly provided by Dr. BZ Carter (University of Texas M. D. Anderson Cancer Center, Houston, TX, USA).^46^ The K562 cell line was obtained from the ATCC. BaF3-p210-WT, BaF3-p210-T315I and K562 cells were cultured in RPMI 1640 medium with 10% FCS as described previously.^41, 47^ The bone marrow cells were obtained from 10 CML patients in the First Affiliated Hospital of Guangzhou Medical University. The research is consistent with the institutional guidelines and the Declaration of Helsinki principles. The isolation of mononuclear cells has been described in previous work.^34, 41^ The cells were suspended in RPMI 1640 supplemented with 15% FCS. All drug treatments were not started until the cells were pre-cultured in fresh medium for 24 h.

### Reagents

PtPT was synthesized in our laboratory and stored as a 20 mM stock solution in dimethyl sulfoxide (DMSO) at −20 °C. Annexin V, propidium iodide (PI) and rhodamine-123 were purchased from Sigma-Aldrich (St. Louis, MO, USA). The proteasome inhibitor, bortezomib (PS341), was purchased from BD Biosciences (San Jose, CA, USA). b-AP15, Suc-LLVY-AMC, Z-LLE-AMC, Boc-LRR-AMC were provided from Boston Biochem (Cambridge, MA, USA). The pan-caspase inhibitor, z-VAD-fmk, was purchased from BD Biosciences. These reagents were dissolved in DMSO as a stock solution, and stored at −20 °C. In all experiments, the final concentration of DMSO did not exceed 0.1%. The metal chelator, ethylenediamine tetraaceticacid (EDTA), was obtained from Sangon Biotech (Shanghai, China). A stored solution (100 mM) was prepared by dissolving EDTA in H_2_O and kept at −20 °C.

### Antibodies

Most of the antibodies were purchased from Cell Signaling Technology (MA, USA) and used at a dilution of 1:1000, including anti-poly adenosine diphophate ribose polymerase (anti-PARP; clone 4C10-5; #9532) and antibodies against phospho-c-Abl at Y245 (#2861), c-Abl (C-19; #2862), phospho-Erk1/2 (T202/Y204; #4370), Erk1/2 (#4348), phospho-Akt (#2965), Akt (#4685), RNA polymerase II (pol II; #2629), phospho-RNA Pol II at Ser2 and Ser5 (#13546), IKB-a (#4814), p27 (#3688), phospho-eIF2a (#9721), eIF2a (#2103), PERK (#5683), Bip (#3177), CHOP (#2895), XIAP (#2045), caspase-8 (#9746), caspase-9 (#9504), apoptosis-inducing factor (AIF) (#5318), Bax (#5023), phospho-STAT5A/B (Y694/Y699; clone 8-5-2; #9314) and STAT5 (#9358). Antibodies against ubiquitin (P4D1; sc-8017), caspase-3 (sc-98785), Bcl-2 (sc-56015), USP14 (SC-515812) and Ki-67 (sc-23900) were from Santa Cruz Biotechnology (Santa Cruz, CA, USA). Antibodies against phospho-USP14 (S241) was from ABGENT. Antibodies against cleaved-caspase-3 (AV00021), cytochrome *c* (C5118) and survivin (S8191) were from Sigma-Aldrich. Anti-GAPDH (#60630) and anti-Actin (#0768) were from Bioworld Technology (St. Louis Park, MN, USA). HRP-conjugated goat anti-rabbit (AP132P) and anti-mouse (12-349) antibodies were from Merck Millipore (Billerica, MA, USA).

### Cell viability assay

Cell viability was measured using MTS assay (CellTiter 96Aqueous One Solution reagent; Promega, Shanghai, China). Briefly, KBM5, KBM5-T315I, K562, BaF3-Bcr/Abl-WT, BaF3-Bcr/Abl-T315I cells were plated in quadruplicate onto the 96-well plates at a density of 2 × 10^4^ cells/ml, then treated with the escalated concentrations of PtPT for 48 h before performing the MTS assay. The absorbance at 490 nm was detected using aplate reader (Varioskan Flash 3001, Thermo, Waltham, MA, USA). The drug concentration that induced 50% inhibition of cell growth (IC_50_) was calculated by regression fitting of a dose-response curve.

### Cell death assay

The CML cells were treated with increasing concentrations of PtPT for durations as indicated in the figures, 0.4% Trypan blue solution was added to monitor temporal changes in the incidence of cell death under the light microscope. Cell apoptosis was evaluated by using of Annexin V/propidium iodide (PI) binding assay according to the instruction of the manufacturer (Sigma-Aldrich). The samples were analyzed using FACSCalibur flow cytometer and CellQuestPro software as previously described.^34, 41^ The Annexin V/PI-positive cells in the culture dish were also imaged with an inverted fluorescence microscope equipped with a digital camera (AxioObsever Z1, Zeiss, Germany).

### Determination of mitochondrial membrane potential

The mitochondrial membrane potential (ΔΨm) of PtPT-treated and untreated cells were measured using rhodamine-123 (Sigma-Aldrich) staining. After being treated with PtPT for 24 h, the cells were incubated with Rh123 (2.5 *μ*g/ml) for further 30 min at 37 °C in the dark. The cells were washed twice with PBS before they were collected for flow cytometry analysis.

### Western blot analysis

Western analyses were performed as previously described.^41, 45^ The whole cell lysates were prepared in RIPA buffer(1 × PBS, 1% NP-40, 0.5% sodiumdeoxycholate, 0.1% SDS) supplemented with freshly added 10 mM *β*-glycerophosphate, 1 mM sodium orthovanadate, 10 mM NaF, 1 mM phenylmethylsulfonyl fluoride, and 1X Roche Complete Mini Protease Inhibitor Cocktail (Roche, Indianapolis, IN, USA). The cytosolic fraction was prepared with digitonin extraction buffer (10 mM PIPES (pH 6.8), 0.015% (wt/vol) digitonin, 300 mM sucrose, 100 mMNaCl, 3 mM MgCl_2_, 5 mM EDTA and 1 mM PMSF) to detect the levels of cytochrome *c* and AIF in the cytosol.

### Proteasomal activity assay

The 20 S proteasomal peptidase activities were measured using synthetic fluorogenic substrates. To evaluate *in vitro* proteasome inhibition, the CML cells were lysed in ice-cold lysis buffer (25 mM Tris-HCl) for 10 min. Equal amounts of protein from each sample were then treated with PtPT, PS341 or b-Ap15 for 30 min, and then incubated at 37 °C with specific fluorogenic substrates (25 *μ*M) for 1–2 h in the dark. The substrates used were Suc-LLVY-AMC for chymotrypsin-like activity, Z-LLE-AMC for caspase-like activity and Boc-LRR-AMC for trypsin-like activity. Fluorescence intensity was measured using a spectrophotometer at excitation of 350 nm and emission of 438 nm (Varioskan Flash 3001, Thermo).

### RNA isolation and reverse transcriptase real-time quantitative polymerase chain reaction (RT^2^-PCR)

Total RNA was extracted by using Trizol reagent (Invitrogen, Carlsbad, CA, USA) as the manufacturer recommended. After quantification by spectrophotometry, the first-strand cDNA was synthesized from 500 ng total RNA using the RNA PCR Kit (AMV) Ver.3.0 (TaKaRa, Dalian, China) and random primers according to the manufacturer’s instructions. RT^2^-PCR was performed using SYBR Premix Ex TaqIIKit (TaKaRa) and Applied Biosystems 7500 Real-Time PCR Systems. The relative gene expression was analyzed by the Comparative Ct method using 18 S ribosomal RNA as the endogenous control. The primers for real-time PCR are as follows: Bcr-Abl forward, 5′-AAGCGCAACAAGCCCACTGTCTAT-3′ reverse, 5′-CTTCGTCTGAGATACTGGATTCCT-3′. 18 S forward, 5′-AAACGGCTACCACATCCAAG-3′ reverse, 5′-CCTCCAATGGATCCTCGTTA-3′.

### Tumor xenograft experiments

The male nu/nu BALB/c mice were bred at the animal facility of Guangzhou Medical University. The mice were housed in barrier facilities with a 12 h light–dark cycle, with food and water available *ad libitum*. KBM5 or KBM5R cells (2 × 10^7^) were inoculated subcutaneously on the flanks of 5- to 6-week-old male nude mice. At 72 h after the inoculation, the mice were treated with either PtPT (7 mg/kg/day) or equivalent volume of the vehicle comprising DMSO, polyethylene glycol 400 and 0.9% NaCl in a 1:3:6 (v:v:v) ratio, for a total of 11 or 14 days. The tumors were measured every other day using calipers. The tumor volumes were calculated with the following formula: *a*^2^ × *b* × 0.4, where *a* is the smallest diameter and *b* is the diameter perpendicular to *a*. The body weight, feeding behavior and motor activity of each animal were monitored as indicators of general health. The animals were then euthanized, and tumor xenografts were immediately removed, weighed, stored and fixed for biochemical or histological analyses. All animal studies were conducted with the approval of the Guangzhou Medical University Institutional Animal Care and Use Committee.

### Immunohistochemical staining

Formalin-fixed xenografts were embedded in paraffin and sectioned using standard techniques. Tumor xenograft sections were immunostained for c-Abl, Ubs, p27 and Ki-67. MaxVision TM reagent (MaixinBiol, Fuzhou, China) was applied to each slide according to the manufacturer’s instructions. The color was developed with 0.05% diaminobenzidine and 0.03% H_2_O_2_ in 50 mmol/l Tris-HCl (pH 7.6), and the slides were counterstained with hematoxylin. A negative control for every antibody was also included for each xenograft specimen by substituting the primary antibody with pre-immune serum.

### Statistical analysis

All the experiments were performed at least thrice, and the results are expressed as mean±95% confidence interval (CI) where applicable. GraphPad Prism 5.0 software was used for statistical analysis. Comparisons between two groups involved two-tailed Student’s *t*-test, and comparisons among multiple groups involved one-way ANOVA with *post hoc* intergroup comparison using Tukey test. *P*-value of <0.05 was considered statistically significant.

## References

[bib1] Rowley JD. Letter: a new consistent chromosomal abnormality in chronic myelogenous leukaemia identified by quinacrine fluorescence and Giemsa staining. Nature 1973; 243: 290–293. 4126434 10.1038/243290a0

[bib2] Druker BJ. Translation of the Philadelphia chromosome into therapy for CML. Blood 2008; 112: 4808–4817. 19064740 10.1182/blood-2008-07-077958

[bib3] Danial NN, Rothman P. JAK-STAT signaling activated by Abl oncogenes. Oncogene 2000; 19: 2523–2531. 10851051 10.1038/sj.onc.1203484

[bib4] Gesbert F, Sellers WR, Signoretti S, Loda M, Griffin JD. BCR/ABL regulates expression of the cyclin-dependent kinase inhibitor p27Kip1 through the phosphatidylinositol 3-Kinase/AKT pathway. J Biol Chem 2000; 275: 39223–39230. 11010972 10.1074/jbc.M007291200

[bib5] Modi H, Li L, Chu S, Rossi J, Yee JK, Bhatia R. Inhibition of Grb2 expression demonstrates an important role in BCR-ABL-mediated MAPK activation and transformation of primary human hematopoietic cells. Leukemia 2011; 25: 305–312. 21072043 10.1038/leu.2010.257 PMC3036781

[bib6] Amarante-Mendes GP, McGahon AJ, Nishioka WK, Afar DE, Witte ON, Green DR. Bcl-2-independent Bcr-Abl-mediated resistance to apoptosis: protection is correlated with upregulation of Bcl-xL. Oncogene 1998; 16: 1383–1390. 9525737 10.1038/sj.onc.1201664

[bib7] Aichberger KJ, Mayerhofer M, Krauth MT, Skvara H, Florian S, Sonneck K et al. Identification of mcl-1 as a BCR/ABL-dependent target in chronic myeloid leukemia (CML): evidence for cooperative antileukemic effects of imatinib and mcl-1 antisense oligonucleotides. Blood 2005; 105: 3303–3311. 15626746 10.1182/blood-2004-02-0749

[bib8] Airiau K, Mahon FX, Josselin M, Jeanneteau M, Turcq B, Belloc F. ABT-737 increases tyrosine kinase inhibitor-induced apoptosis in chronic myeloid leukemia cells through XIAP downregulation and sensitizes CD34(+) CD38(-) population to imatinib. Exp Hematol 2012; 40: 367–78.e2. 22240609 10.1016/j.exphem.2012.01.004

[bib9] Schindler T, Bornmann W, Pellicena P, Miller WT, Clarkson B, Kuriyan J. Structural mechanism for STI-571 inhibition of abelson tyrosine kinase. Science 2000; 289: 1938–1942. 10988075 10.1126/science.289.5486.1938

[bib10] Sawyers CL, Hochhaus A, Feldman E, Goldman JM, Miller CB, Ottmann OG et al. Imatinib induces hematologic and cytogenetic responses in patients with chronic myelogenous leukemia in myeloid blast crisis: results of a phase II study. Blood 2002; 99: 3530–3539. 11986204 10.1182/blood.v99.10.3530

[bib11] Talpaz M, Silver RT, Druker BJ, Goldman JM, Gambacorti-Passerini C, Guilhot F et al. Imatinib induces durable hematologic and cytogenetic responses in patients with accelerated phase chronic myeloid leukemia: results of a phase 2 study. Blood 2002; 99: 1928–1937. 11877262 10.1182/blood.v99.6.1928

[bib12] Kantarjian HM, Talpaz M, Giles F, O'Brien S, Cortes J. New insights into the pathophysiology of chronic myeloid leukemia and imatinib resistance. Ann Intern Med 2006; 145: 913–923. 17179059 10.7326/0003-4819-145-12-200612190-00008

[bib13] Balabanov S, Braig M, Brummendorf TH. Current aspects in resistance against tyrosine kinase inhibitors in chronic myelogenous leukemia. Drug Discov Today Technol 2014; 11: 89–99. 24847658 10.1016/j.ddtec.2014.03.003

[bib14] Gambacorti-Passerini CB, Gunby RH, Piazza R, Galietta A, Rostagno R, Scapozza L. Molecular mechanisms of resistance to imatinib in Philadelphia-chromosome-positive leukaemias. Lancet Oncol 2003; 4: 75–85. 12573349 10.1016/s1470-2045(03)00979-3

[bib15] Apperley JF. Part I: mechanisms of resistance to imatinib in chronic myeloid leukaemia. Lancet Oncol 2007; 8: 1018–1029. 17976612 10.1016/S1470-2045(07)70342-X

[bib16] Branford S. Chronic myeloid leukemia: molecular monitoring in clinical practice. Hematology Am Soc Hematol Educ Program 2007: 376–383. 18024654 10.1182/asheducation-2007.1.376

[bib17] Vaidya S, Ghosh K, Vundinti BR. Recent developments in drug resistance mechanism in chronic myeloid leukemia: a review. Eur J Haematol 2011; 87: 381–393. 21815933 10.1111/j.1600-0609.2011.01689.x

[bib18] O'Hare T, Eide CA, Deininger MW. Bcr-Abl kinase domain mutations, drug resistance, and the road to a cure for chronic myeloid leukemia. Blood 2007; 110: 2242–2249. 17496200 10.1182/blood-2007-03-066936

[bib19] Talpaz M, Shah NP, Kantarjian H, Donato N, Nicoll J, Paquette R et al. Dasatinib in imatinib-resistant Philadelphia chromosome-positive leukemias. N Engl J Med 2006; 354: 2531–2541. 16775234 10.1056/NEJMoa055229

[bib20] Kaur P, Feldhahn N, Zhang B, Trageser D, Muschen M, Pertz V et al. Nilotinib treatment in mouse models of P190 Bcr/Abl lymphoblastic leukemia. Mol Cancer 2007; 6: 67. 17958915 10.1186/1476-4598-6-67 PMC2169263

[bib21] Redaelli S, Piazza R, Rostagno R, Magistroni V, Perini P, Marega M et al. Activity of bosutinib, dasatinib, and nilotinib against 18 imatinib-resistant BCR/ABL mutants. J Clin Oncol 2009; 27: 469–471. 19075254 10.1200/JCO.2008.19.8853

[bib22] Wehrle J, Pahl HL, von Bubnoff N. Ponatinib: a third-generation inhibitor for the treatment of CML. Recent Results Cancer Res 2014; 201: 99–107. 24756787 10.1007/978-3-642-54490-3_5

[bib23] Zabriskie MS, Eide CA, Tantravahi SK, Vellore NA, Estrada J, Nicolini FE et al. BCR-ABL1 compound mutations combining key kinase domain positions confer clinical resistance to ponatinib in Ph chromosome-positive leukemia. Cancer Cell 2014; 26: 428–442. 25132497 10.1016/j.ccr.2014.07.006 PMC4160372

[bib24] Richardson PG, Barlogie B, Berenson J, Singhal S, Jagannath S, Irwin D et al. A phase 2 study of bortezomib in relapsed, refractory myeloma. N Engl J Med 2003; 348: 2609–2617. 12826635 10.1056/NEJMoa030288

[bib25] Zhang L, Pham LV, Newberry KJ, Ou Z, Liang R, Qian J et al. *In vitro* and *in vivo* therapeutic efficacy of carfilzomib in mantle cell lymphoma: targeting the immunoproteasome. Mol Cancer Ther 2013; 12: 2494–2504. 23990113 10.1158/1535-7163.MCT-13-0156

[bib26] Komander D, Clague MJ, Urbe S. Breaking the chains: structure and function of the deubiquitinases. Nat Rev Mol Cell Biol 2009; 10: 550–563. 19626045 10.1038/nrm2731

[bib27] Fraile JM, Quesada V, Rodriguez D, Freije JM, Lopez-Otin C. Deubiquitinases in cancer: new functions and therapeutic options. Oncogene 2012; 31: 2373–2388. 21996736 10.1038/onc.2011.443

[bib28] Yan M, Luo JK, Ritchie KJ, Sakai I, Takeuchi K, Ren R et al. Ubp43 regulates BCR-ABL leukemogenesis via the type 1 interferon receptor signaling. Blood 2007; 110: 305–312. 17374743 10.1182/blood-2006-07-033209 PMC1896118

[bib29] Hussain S, Zhang Y, Galardy PJ. DUBs and cancer: the role of deubiquitinating enzymes as oncogenes, non-oncogenes and tumor suppressors. Cell Cycle 2009; 8: 1688–1697. 19448430 10.4161/cc.8.11.8739

[bib30] Lawson AP, Long MJ, Coffey RT, Qian Y, Weerapana E, El Oualid F et al. Naturally occurring isothiocyanates exert anticancer effects by inhibiting deubiquitinating enzymes. Cancer Res 2015; 75: 5130–5142. 26542215 10.1158/0008-5472.CAN-15-1544 PMC4668232

[bib31] Peth A, Kukushkin N, Bosse M, Goldberg AL. Ubiquitinated proteins activate the proteasomal ATPases by binding to Usp14 or Uch37 homologs. J Biol Chem 2013; 288: 7781–7790. 23341450 10.1074/jbc.M112.441907 PMC3597817

[bib32] D'Arcy P, Linder S. Molecular pathways: translational potential of deubiquitinases as drug targets. Clin Cancer Res 2014; 20: 3908–3914. 25085788 10.1158/1078-0432.CCR-14-0568

[bib33] Heaney NB, Pellicano F, Zhang B, Crawford L, Chu S, Kazmi SM et al. Bortezomib induces apoptosis in primitive chronic myeloid leukemia cells including LTC-IC and NOD/SCID repopulating cells. Blood 2010; 115: 2241–2250. 20068223 10.1182/blood-2008-06-164582 PMC2844011

[bib34] Shi X, Chen X, Li X, Lan X, Zhao C, Liu S et al. Gambogic acid induces apoptosis in imatinib-resistant chronic myeloid leukemia cells via inducing proteasome inhibition and caspase-dependent Bcr-Abl downregulation. Clin Cancer Res 2014; 20: 151–163. 24334603 10.1158/1078-0432.CCR-13-1063 PMC3938960

[bib35] Zhao C, Chen X, Zang D, Lan X, Liao S, Yang C et al. Platinum-containing compound platinum pyrithione is stronger and safer than cisplatin in cancer therapy. Biochem Pharmacol 2016; 116: 22–38. 27381943 10.1016/j.bcp.2016.06.019 PMC5287571

[bib36] Selvaraju K, Mazurkiewicz M, Wang X, Gullbo J, Linder S, D'Arcy P. Inhibition of proteasome deubiquitinase activity: a strategy to overcome resistance to conventional proteasome inhibitors? Drug Resist Updat 2015; 21-22: 20–29. 26183292 10.1016/j.drup.2015.06.001

[bib37] Xu D, Shan B, Lee BH, Zhu K, Zhang T, Sun H et al. Phosphorylation and activation of ubiquitin-specific protease-14 by Akt regulates the ubiquitin-proteasome system. Elife 2015; 4: e10510. 26523394 10.7554/eLife.10510 PMC4733041

[bib38] Kao C, Chao A, Tsai CL, Chuang WC, Huang WP, Chen GC et al. Bortezomib enhances cancer cell death by blocking the autophagic flux through stimulating ERK phosphorylation. Cell Death Dis 2014; 5: e1510. 25375375 10.1038/cddis.2014.468 PMC4260726

[bib39] Hisatomi T, Nakao S, Murakami Y, Noda K, Nakazawa T, Notomi S et al. The regulatory roles of apoptosis-inducing factor in the formation and regression processes of ocular neovascularization. Am J Pathol 2012; 181: 53–61. 22613025 10.1016/j.ajpath.2012.03.022 PMC3388154

[bib40] Menendez-Benito V, Verhoef LG, Masucci MG, Dantuma NP. Endoplasmic reticulum stress compromises the ubiquitin-proteasome system. Hum Mol Genet 2005; 14: 2787–2799. 16103128 10.1093/hmg/ddi312

[bib41] Shi X, Jin Y, Cheng C, Zhang H, Zou W, Zheng Q et al. Triptolide inhibits Bcr-Abl transcription and induces apoptosis in STI571-resistant chronic myelogenous leukemia cells harboring T315I mutation. Clin Cancer Res 2009; 15: 1686–1697. 19240172 10.1158/1078-0432.CCR-08-2141

[bib42] O'Hare T, Zabriskie MS, Eide CA, Agarwal A, Adrian LT, You H et al. The BCR-ABL35INS insertion/truncation mutant is kinase-inactive and does not contribute to tyrosine kinase inhibitor resistance in chronic myeloid leukemia. Blood 2011; 118: 5250–5254. 21908430 10.1182/blood-2011-05-349191 PMC3217407

[bib43] Huang H, Liao Y, Liu N, Hua X, Cai J, Yang C et al. Two clinical drugs deubiquitinase inhibitor auranofin and aldehyde dehydrogenase inhibitor disulfiram trigger synergistic anti-tumor effects *in vitro* and *in vivo*. Oncotarget 2016; 7: 2796–2808. 26625200 10.18632/oncotarget.6425 PMC4823072

[bib44] Ben-Neriah Y, Daley GQ, Mes-Masson AM, Witte ON, Baltimore D. The chronic myelogenous leukemia-specific P210 protein is the product of the bcr/abl hybrid gene. Science 1986; 233: 212–214. 3460176 10.1126/science.3460176

[bib45] Chen X, Shi X, Zhao C, Li X, Lan X, Liu S et al. Anti-rheumatic agent auranofin induced apoptosis in chronic myeloid leukemia cells resistant to imatinib through both Bcr/Abl-dependent and -independent mechanisms. Oncotarget 2014; 5: 9118–9132. 25193854 10.18632/oncotarget.2361 PMC4253423

[bib46] Mak DH, Schober WD, Chen W, Konopleva M, Cortes J, Kantarjian HM et al. Triptolide induces cell death independent of cellular responses to imatinib in blast crisis chronic myelogenous leukemia cells including quiescent CD34+ primitive progenitor cells. Mol Cancer Ther 2009; 8: 2509–2516. 19723894 10.1158/1535-7163.MCT-09-0386 PMC2754862

[bib47] Carter BZ, Mak DH, Schober WD, Cabreira-Hansen M, Beran M, McQueen T et al. Regulation of survivin expression through Bcr-Abl/MAPK cascade: targeting survivin overcomes imatinib resistance and increases imatinib sensitivity in imatinib-responsive CML cells. Blood 2006; 107: 1555–1563. 16254145 10.1182/blood-2004-12-4704 PMC1895411

